# Supplementary figures and images for: Roles of lung-recruited monocytes and pulmonary Vascular Endothelial Growth Factor (VEGF) in resolving Ventilator-Induced Lung Injury (VILI)
Source: PLoS One. 2021 Mar 19;16(3):e0248959. doi: 10.1371/journal.pone.0248959 (PMC7978382; doi:10.1371/journal.pone.0248959)

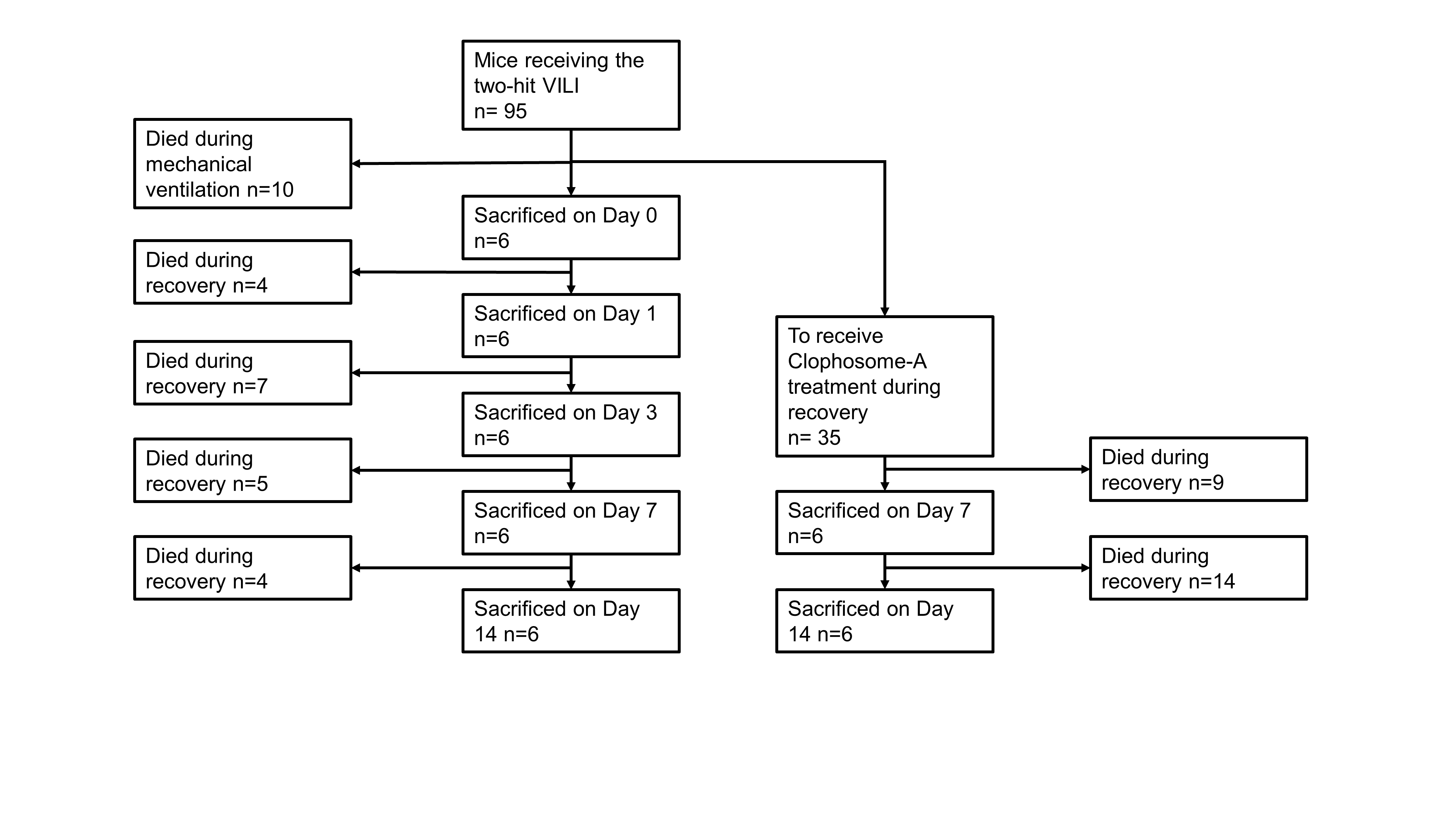

Supplement: S1 Fig — The numbers of animals used in the different experimental groups and the numbers of animals died during mechanical ventilation and recovery. (TIF) [file pone.0248959.s001.tif]
