# Supplementary material for: Roles of lung-recruited monocytes and pulmonary Vascular Endothelial Growth Factor (VEGF) in resolving Ventilator-Induced Lung Injury (VILI)
Source: PLoS One. 2021 Mar 19;16(3):e0248959. doi: 10.1371/journal.pone.0248959 (PMC7978382; doi:10.1371/journal.pone.0248959)
Supplement: S1 Text — (DOCX) [file pone.0248959.s002.docx]

Detailed illustrations of the statistical analysis

**Fig 1. page 1-3**

**Fig 2. page 4-9**

**Fig 3. page 10-20**

**Fig 4. page 21-25**

**Fig 5. page 26-28**

**Fig 6. page 29-30**

**Fig 1. (B) Quantification and comparison of the inflammatory cells in lung sections from individual mice sacrificed at different time points.**

| **Tests of Normality** | | | | | | | | | | | | | | | |
| --- | --- | --- | --- | --- | --- | --- | --- | --- | --- | --- | --- | --- | --- | --- | --- |
|  | | | Kolmogorov-Smirnov | | | | | | | Shapiro-Wilk | | | | | |
|  |  |  | Statistic | | df | | | Sig. | | Statistic | | df | | Sig. | |
| Inflammtory cells/100 alveoli | | | 0.128 | | 21 | | | 0.200^*^ | | 0.924 | | 21 | | 0.104 | |
|  | | | | | | | | | | | | | | | |
| **Descriptives** | | | | | | | | | | | | | | |  |
| Inflammatory cells/100 alveoli | | | | | | | | | | | | | | |  |
| Day after VIL | N | Mean | | Std. Deviation | | Std. Error | 95% Confidence Interval for Mean | | | | Minimum | | Maximum | |  |
|  |  |  |  |  |  |  | Lower Bound | | Upper Bound | |  |  |  |  |  |
| 0 | 3 | 45 | | 3 | | 2 | 38 | | 52 | | 42 | | 48 | |  |
| 1 | 3 | 53 | | 1 | | 1 | 51 | | 55 | | 52 | | 54 | |  |
| 3 | 3 | 39 | | 4 | | 2 | 29 | | 49 | | 35 | | 43 | |  |
| 7 | 5 | 25 | | 1 | | 1 | 23 | | 26 | | 23 | | 26 | |  |
| 14 | 3 | 14 | | 2 | | 1 | 9 | | 19 | | 12 | | 16 | |  |
| C | 4 | 6 | | 1 | | 1 | 3 | | 8 | | 4 | | 7 | |  |
| Total | 21 | 28 | | 17 | | 4 | 21 | | 36 | | 4 | | 54 | |  |

C: control

| **Test of Homogeneity of Variances** | | | |
| --- | --- | --- | --- |
| Inflammatory cells/100 alveoli | | | |
| Levene Statistic | df1 | df2 | Sig. |
| 1.298 | 5 | 15 | 0.316 |

| **ANOVA** | | | | | |
| --- | --- | --- | --- | --- | --- |
| Inflammatory cells/100 alveoli | | | | | |
|  | Sum of Squares | df | Mean Square | F | Sig. |
| Between Groups | 5774.952 | 5 | 1154.990 | 247.498 | <0.001 |
| Within Groups | 70.000 | 15 | 4.667 |  |  |
| Total | 5844.952 | 20 |  |  |  |

| **Multiple Comparisons** | | | | | | | | | |
| --- | --- | --- | --- | --- | --- | --- | --- | --- | --- |
| Dependent Variable: Inflammatory cells/100 alveoli | | | | | | | | | |
|  | (I) day after VILI | | (J) day after VILI | | Mean Difference (I-J) | Std. Error | Sig. | 95% Confidence Interval | |
|  |  |  |  |  |  |  |  | Lower Bound | Upper Bound |
| Bonferroni |  | 0 |  | 1 | -8 | 1.764 | 0.006 | -14.14 | -1.86 |
|  |  |  |  | 3 | 6 | 1.764 | 0.059 | -0.14 | 12.14 |
|  |  |  |  | 7 | 20.5 | 1.578 | <0.001 | 15 | 26 |
|  |  |  |  | 14 | 31 | 1.764 | <0.001 | 24.86 | 37.14 |
|  |  |  |  | C | 39.5 | 1.65 | <0.001 | 33.75 | 45.25 |
|  |  | 1 |  | 0 | 8 | 1.764 | 0.006 | 1.86 | 14.14 |
|  |  |  |  | 3 | 14 | 1.764 | <0.001 | 7.86 | 20.14 |
|  |  |  |  | 7 | 28.5 | 1.578 | <0.001 | 23 | 34 |
|  |  |  |  | 14 | 39 | 1.764 | <0.001 | 32.86 | 45.14 |
|  |  |  |  | C | 47.5 | 1.65 | <0.001 | 41.75 | 53.25 |
|  |  | 3 |  | 0 | -6 | 1.764 | 0.059 | -12.14 | 0.14 |
|  |  |  |  | 1 | -14 | 1.764 | <0.001 | -20.14 | -7.86 |
|  |  |  |  | 7 | 14.5 | 1.578 | <0.001 | 9 | 20 |
|  |  |  |  | 14 | 25 | 1.764 | <0.001 | 18.86 | 31.14 |
|  |  |  |  | C | 33.5 | 1.65 | <0.001 | 27.75 | 39.25 |
|  |  | 7 |  | 0 | -20.5 | 1.578 | <0.001 | -26 | -15 |
|  |  |  |  | 1 | -28.5 | 1.578 | <0.001 | -34 | -23 |
|  |  |  |  | 3 | -14.5 | 1.578 | <0.001 | -20 | -9 |
|  |  |  |  | 14 | 10.5 | 1.578 | <0.001 | 5 | 16 |
|  |  |  |  | C | 19 | 1.449 | <0.001 | 13.95 | 24.05 |
|  |  | 14 |  | 0 | -31 | 1.764 | <0.001 | -37.14 | -24.86 |
|  |  |  |  | 1 | -39 | 1.764 | <0.001 | -45.14 | -32.86 |
|  |  |  |  | 3 | -25 | 1.764 | <0.001 | -31.14 | -18.86 |
|  |  |  |  | 7 | -10.5 | 1.578 | <0.001 | -16 | -5 |
|  |  |  |  | C | 8.5 | 1.65 | 0.002 | 2.75 | 14.25 |
|  |  | C |  | 0 | -39.5 | 1.65 | <0.001 | -45.25 | -33.75 |
|  |  |  |  | 1 | -47.5 | 1.65 | <0.001 | -53.25 | -41.75 |
|  |  |  |  | 3 | -33.5 | 1.65 | <0.001 | -39.25 | -27.75 |
|  |  |  |  | 7 | -19 | 1.449 | <0.001 | -24.05 | -13.95 |
|  |  |  |  | 14 | -8.5 | 1.65 | 0.002 | -14.25 | -2.75 |

C: control

**Fig 2. (C) Quantification and comparison of lung-recruited CD11b^+^ leukocytes of individual mice sacrificed at different time points.**

| **Tests of Normality** | | | | | | | | | | | | | | |
| --- | --- | --- | --- | --- | --- | --- | --- | --- | --- | --- | --- | --- | --- | --- |
|  | | | Kolmogorov-Smirnov | | | | | | Shapiro-Wilk | | | | | |
|  |  |  | Statistic | | df | | Sig. | | Statistic | | df | | Sig. | |
| total CD11b | | | 0.399 | | 26 | | <0.001 | | 0.650 | | 26 | | <0.001 | |
|  | | | | | | | | | | | | | | |
| **Descriptives** | | | | | | | | | | | | | | |
| total CD11b^+^ | | | | | | | | | | | | | | |
| Day | N | Mean (cell number) | | Std. Deviation | | Std. Error | | 95% Confidence Interval for Mean | | | | Minimum | | Maximum |
|  |  |  |  |  |  |  |  | Lower Bound | | Upper Bound | |  |  |  |
| 0 | 4 | 3522225 | | 1365439.4 | | 682719.7 | | 1349506.2 | | 5694943.8 | | 1748000 | | 4944000 |
| 1 | 4 | 4506288 | | 630851.6 | | 315425.8 | | 3502461.8 | | 5510113.2 | | 3600000 | | 5022400 |
| 3 | 4 | 97139 | | 40189.0 | | 20094.5 | | 33189.1 | | 161088.4 | | 64960 | | 154905 |
| 7 | 5 | 102668 | | 79059.7 | | 35356.6 | | 4502.5 | | 200833.5 | | 38340 | | 230400 |
| 14 | 5 | 65818 | | 29042.2 | | 12988.1 | | 29757.4 | | 101878.6 | | 15960 | | 88650 |
| C | 4 | 28175 | | 16896.3 | | 8448.1 | | 1289.0 | | 55060.5 | | 15744 | | 52864 |
| Total | 26 | 1286836 | | 1946614.8 | | 381762.6 | | 500581.2 | | 2073090.6 | | 15744 | | 5022400 |

C: control

| **Independent-Samples Kruskal-Wallis Test Summary** | |
| --- | --- |
| Total N | 26 |
| Test Statistic | 19.497^a^ |
| Degree Of Freedom | 5 |
| Asymptotic Sig.(2-sided test) | 0.002 |
| a. The test statistic is adjusted for ties. | |

| **Pairwise Comparisons of day after VILI** | | | | | |
| --- | --- | --- | --- | --- | --- |
| Sample 1-Sample 2 | Test Statistic | Std. Error | Std. Test Statistic | Sig. | Adj. Sig.^a^ |
| C-Day14 | 5.650 | 5.131 | 1.101 | 0.271 | 1.000 |
| C-Day 7 | 7.850 | 5.131 | 1.530 | 0.126 | 1.000 |
| C- Day 3 | 9.000 | 5.408 | 1.664 | 0.096 | 1.000 |
| C- Day 0 | 17.750 | 5.408 | 3.282 | 0.001 | 0.022 |
| C- Day 1 | 19.750 | 5.408 | 3.652 | <0.001 | 0.005 |
| Day 14- Day 7 | -2.200 | 4.837 | -0.455 | 0.649 | 1.000 |
| Day 14- Day 3 | -3.350 | 5.131 | -0.653 | 0.514 | 1.000 |
| Day 14- Day 0 | 12.100 | 5.131 | 2.358 | 0.018 | 0.386 |
| Day 14- Day 1 | 14.100 | 5.131 | 2.748 | 0.006 | 0.126 |
| Day 7- Day 3 | 1.150 | 5.131 | 0.224 | 0.823 | 1.000 |
| Day 7- Day 0 | 9.900 | 5.131 | 1.930 | 0.054 | 1.000 |
| Day 7- Day 1 | 11.900 | 5.131 | 2.319 | 0.020 | 0.428 |
| Day 3- Day 0 | 8.750 | 5.408 | 1.618 | 0.106 | 1.000 |
| Day 3- Day 1 | 10.750 | 5.408 | 1.988 | 0.047 | 0.984 |
| Day 0- Day 1 | -2.000 | 5.408 | -0.370 | 0.712 | 1.000 |
| Each row tests the null hypothesis that the Sample 1 and Sample 2 distributions are the same.  Asymptotic significances (2-sided tests) are displayed. The significance level is 0.05. | | | | | |
| a. Significance values have been adjusted by the Bonferroni correction for multiple tests. | | | | | |

C: control

**Fig 2. (D) The percentages of pulmonary Ly6C^+high^ and Ly6C^+low^ monocytes of individual mice sacrificed at different time points.**

| **Tests of Normality** | | | | | | |
| --- | --- | --- | --- | --- | --- | --- |
|  | Kolmogorov-Smirnov | | | Shapiro-Wilk | | |
|  | Statistic | df | Sig. | Statistic | df | Sig. |
| Ly6C+high F4/80+ | 0.204 | 26 | 0.007 | 0.936 | 26 | 0.110 |
|  | | | | | | |

| **Tests of Normality** | | | | | | |
| --- | --- | --- | --- | --- | --- | --- |
|  | Kolmogorov-Smirnov | | | Shapiro-Wilk | | |
|  | Statistic | df | Sig. | Statistic | df | Sig. |
| Ly6C+low F4/80+ | 0.204 | 26 | 0.007 | .936 | 26 | 0.110 |
|  | | | | | | |

| **Descriptives** | | | | | | | | | |
| --- | --- | --- | --- | --- | --- | --- | --- | --- | --- |
|  | Day after VILI | N | Mean (%) | Std. Deviation | Std. Error | 95% Confidence Interval for Mean | | Minimum | Maximum |
|  |  |  |  |  |  | Lower Bound | Upper Bound |  |  |
| Ly6C^+high^ F4/80^+^ | 0 | 4 | 62.2 | 7.3 | 3.7 | 50.6 | 73.9 | 55.3 | 72.5 |
|  | 1 | 4 | 28.3 | 6.9 | 3.5 | 17.3 | 39.3 | 20.3 | 35.7 |
|  | 3 | 4 | 26.4 | 9.8 | 4.9 | 10.8 | 42.0 | 14.3 | 34.8 |
|  | 7 | 5 | 35.6 | 8.9 | 4.0 | 24.6 | 46.6 | 24.0 | 45.3 |
|  | 14 | 5 | 28.3 | 2.9 | 1.3 | 24.7 | 31.9 | 25.1 | 32.9 |
|  | C | 4 | 18.5 | 12.6 | 6.3 | -1.5 | 38.6 | 6.1 | 34.8 |
|  | Total | 26 | 33.1 | 15.6 | 3.1 | 26.8 | 39.4 | 6.1 | 72.5 |
| Ly6C^+low^ F4/80^+^ | 0 | 4 | 37.8 | 7.3 | 3.7 | 26.1 | 49.4 | 27.5 | 44.7 |
|  | 1 | 4 | 71.7 | 6.9 | 3.5 | 60.7 | 82.7 | 64.3 | 79.7 |
|  | 3 | 4 | 73.6 | 9.8 | 4.9 | 58.0 | 89.2 | 65.2 | 85.7 |
|  | 7 | 5 | 64.4 | 8.9 | 4.0 | 53.4 | 75.4 | 54.7 | 76.0 |
|  | 14 | 5 | 71.7 | 2.9 | 1.3 | 68.1 | 75.3 | 67.1 | 74.9 |
|  | C | 4 | 81.5 | 12.6 | 6.3 | 61.4 | 101.5 | 65.2 | 93.9 |
|  | Total | 26 | 66.9 | 15.6 | 3.1 | 60.6 | 73.2 | 27.5 | 93.9 |

C: control

| **Test of Homogeneity of Variances** | | | | |
| --- | --- | --- | --- | --- |
|  | Levene Statistic | df1 | df2 | Sig. |
| Ly6C^+high^ F4/80^+^ | 2.144 | 5 | 20 | 0.102 |
| Ly6C^+low^ F4/80^+^ | 2.144 | 5 | 20 | 0.102 |

| **ANOVA** | | | | | | |
| --- | --- | --- | --- | --- | --- | --- |
|  | | Sum of Squares | df | Mean Square | F | Sig. |
| Ly6C^+high^ F4/80^+^ | Between Groups | 4664.905 | 5 | 932.981 | 13.152 | <0.001 |
|  | Within Groups | 1418.787 | 20 | 70.939 |  |  |
|  | Total | 6083.692 | 25 |  |  |  |
| Ly6C^+low^ F4/80^+^ | Between Groups | 4664.905 | 5 | 932.981 | 13.152 | <0.001 |
|  | Within Groups | 1418.787 | 20 | 70.939 |  |  |
|  | Total | 6083.692 | 25 |  |  |  |

| **Multiple Comparisons** | | | | | | | | |
| --- | --- | --- | --- | --- | --- | --- | --- | --- |
| Dependent Variable | | (I) day after VILI | (J) day after VILI | Mean Difference (I-J) | Std. Error | Sig. | 95% Confidence Interval | |
|  |  |  |  |  |  |  | Lower Bound | Upper Bound |
| Ly6C+low F4/80+ | Bonferroni | 0 | 1 | -33.966 | 5.956 | <0.001 | -53.80 | -14.13 |
|  |  |  | 3 | -35.864 | 5.956 | <0.001 | -55.70 | -16.03 |
|  |  |  | 7 | -26.660 | 5.650 | 0.002 | -45.48 | -7.84 |
|  |  |  | 14 | -33.952 | 5.650 | <0.001 | -52.77 | -15.13 |
|  |  |  | C | -43.699 | 5.956 | <0.001 | -63.53 | -23.86 |
|  |  | 1 | 0 | 33.966 | 5.956 | <0.001 | 14.13 | 53.80 |
|  |  |  | 3 | -1.898 | 5.956 | 1.000 | -21.73 | 17.94 |
|  |  |  | 7 | 7.306 | 5.650 | 1.000 | -11.51 | 26.12 |
|  |  |  | 14 | 0.013 | 5.650 | 1.000 | -18.80 | 18.83 |
|  |  |  | C | -9.733 | 5.956 | 1.000 | -29.57 | 10.10 |
|  |  | 3 | 0 | 35.864 | 5.956 | <0.001 | 16.03 | 55.70 |
|  |  |  | 1 | 1.898 | 5.956 | 1.000 | -17.94 | 21.73 |
|  |  |  | 7 | 9.204 | 5.650 | 1.000 | -9.61 | 28.02 |
|  |  |  | 14 | 1.912 | 5.650 | 1.000 | -16.91 | 20.73 |
|  |  |  | C | -7.835 | 5.956 | 1.000 | -27.67 | 12.00 |
|  |  | 7 | 0 | 26.660 | 5.650 | 0.002 | 7.84 | 45.48 |
|  |  |  | 1 | -7.306 | 5.650 | 1.000 | -26.12 | 11.51 |
|  |  |  | 3 | -9.204 | 5.650 | 1.000 | -28.02 | 9.61 |
|  |  |  | 14 | -7.292 | 5.327 | 1.000 | -25.03 | 10.45 |
|  |  |  | C | -17.039 | 5.650 | 0.102 | -35.86 | 1.78 |
|  |  | 14 | 0 | 33.952 | 5.650 | <0.001 | 15.13 | 52.77 |
|  |  |  | 1 | -0.013 | 5.650 | 1.000 | -18.83 | 18.80 |
|  |  |  | 3 | -1.912 | 5.650 | 1.000 | -20.73 | 16.91 |
|  |  |  | 7 | 7.292 | 5.327 | 1.000 | -10.45 | 25.03 |
|  |  |  | C | -9.747 | 5.650 | 1.000 | -28.56 | 9.07 |
|  |  | C | 0 | 43.699 | 5.956 | <0.001 | 23.86 | 63.53 |
|  |  |  | 1 | 9.733 | 5.956 | 1.000 | -10.10 | 29.57 |
|  |  |  | 3 | 7.835 | 5.956 | 1.000 | -12.00 | 27.67 |
|  |  |  | 7 | 17.039 | 5.650 | 0.102 | -1.78 | 35.86 |
|  |  |  | 14 | 9.747 | 5.650 | 1.000 | -9.07 | 28.56 |

C: control

**Fig 3.** **ELISA for inflammatory cytokines and growth factors in the lung tissue, (A) IL-6**

| **Tests of Normality** | | | | | | |
| --- | --- | --- | --- | --- | --- | --- |
|  | Kolmogorov-Smirnov^a^ | | | Shapiro-Wilk | | |
|  | Statistic | df | Sig. | Statistic | df | Sig. |
| IL-6 pg/g | 0.275 | 28 | <0.001 | 0.766 | 28 | <0.001 |
| a. Lilliefors Significance Correction | | | | | | |

| **Descriptives** | | | | | | | | |
| --- | --- | --- | --- | --- | --- | --- | --- | --- |
| IL-6 pg/g | | | | | | | | |
| Day after VILI | N | Mean | Std. Deviation | Std. Error | 95% Confidence Interval for Mean | | Minimum | Maximum |
|  |  |  |  |  | Lower Bound | Upper Bound |  |  |
| 0 | 4 | 15817.5 | 703.5 | 351.8 | 14698.0 | 16937.0 | 14772.0 | 16293.0 |
| 1 | 4 | 15279.0 | 2232.5 | 1116.2 | 11726.6 | 18831.4 | 12192.0 | 17517.0 |
| 3 | 4 | 7170.8 | 151.3 | 75.6 | 6930.0 | 7411.5 | 7014.0 | 7371.0 |
| 7 | 6 | 1414.9 | 900.9 | 367.8 | 469.5 | 2360.3 | 366.0 | 2400.0 |
| 14 | 6 | 399.5 | 158.0 | 64.5 | 233.7 | 565.3 | 261.0 | 599.8 |
| C | 4 | 586.2 | 189.6 | 94.8 | 284.6 | 887.8 | 387.0 | 762.0 |
| Total | 28 | 5939.3 | 6632.4 | 1253.4 | 3367.5 | 8511.0 | 261.0 | 17517.0 |

C: control

| **Independent-Samples Kruskal-Wallis Test Summary** | |
| --- | --- |
| Total N | 28 |
| Test Statistic | 23.936^a^ |
| Degree Of Freedom | 5 |
| Asymptotic Sig.(2-sided test) | <0.001 |
| a. The test statistic is adjusted for ties. | |

| **Pairwise Comparisons of Recovery day** | | | | | |
| --- | --- | --- | --- | --- | --- |
| Sample 1-Sample 2 | Test Statistic | Std. Error | Std. Test Statistic | Sig. | Adj. Sig.^a^ |
| Day 14-C | 4.500 | 5.310 | 0.847 | 0.397 | 1.000 |
| Day 14-Day 7 | -7.667 | 4.749 | -1.614 | 0.106 | 1.000 |
| Day 14-Day 3 | -14.000 | 5.310 | -2.637 | 0.008 | 0.176 |
| Day 14-Day 1 | 19.500 | 5.310 | 3.672 | <0.001 | 0.005 |
| Day 14-Day 0 | 20.500 | 5.310 | 3.861 | <0.001 | 0.002 |
| C-Day 7 | -3.167 | 5.310 | -0.596 | 0.551 | 1.000 |
| C-Day 3 | -9.500 | 5.817 | -1.633 | 0.102 | 1.000 |
| C-Day 1 | -15.000 | 5.817 | -2.579 | 0.010 | 0.208 |
| C-Day 0 | -16.000 | 5.817 | -2.751 | 0.006 | 0.125 |
| Day 7-Day 3 | 6.333 | 5.310 | 1.193 | 0.233 | 1.000 |
| Day 7-Day 1 | 11.833 | 5.310 | 2.229 | 0.026 | 0.543 |
| Day 7-Day 0 | 12.833 | 5.310 | 2.417 | 0.016 | 0.329 |
| Day 3-Day 1 | 5.500 | 5.817 | 0.946 | 0.344 | 1.000 |
| Day 3-Day 0 | 6.500 | 5.817 | 1.117 | 0.264 | 1.000 |
| Day 1-Day 0 | 1.000 | 5.817 | 0.172 | 0.864 | 1.000 |
| Each row tests the null hypothesis that the Sample 1 and Sample 2 distributions are the same.  Asymptotic significances (2-sided tests) are displayed. The significance level is 0.05. | | | | | |
| a. Significance values have been adjusted by the Bonferroni correction for multiple tests. | | | | | |

C=control

**Fig 3. ELISA for inflammatory cytokines and growth factors in the lung tissue, (B) IL-1β**

| **Tests of Normality** | | | | | | |
| --- | --- | --- | --- | --- | --- | --- |
|  | Kolmogorov-Smirnov^a^ | | | Shapiro-Wilk | | |
|  | Statistic | df | Sig. | Statistic | df | Sig. |
| IL-1b pg/g | 0.139 | 28 | 0.180 | 0.879 | 28 | 0.004 |
| a. Lilliefors Significance Correction | | | | | | |

| **Descriptives** | | | | | | | | |
| --- | --- | --- | --- | --- | --- | --- | --- | --- |
| IL-1b pg/g | | | | | | | | |
| Day after VILI | N | Mean | Std. Deviation | Std. Error | 95% Confidence Interval for Mean | | Minimum | Maximum |
|  |  |  |  |  | Lower Bound | Upper Bound |  |  |
| 0 | 4 | 7486.4 | 1754.3 | 877.1 | 4695.0 | 10277.9 | 5231.6 | 9512.8 |
| 1 | 4 | 8861.8 | 517.9 | 258.9 | 8037.7 | 9685.8 | 8433.3 | 9477.8 |
| 3 | 4 | 3815.5 | 1147.2 | 573.6 | 1990.1 | 5640.8 | 2244.4 | 4937.5 |
| 7 | 6 | 2828.7 | 1827.3 | 746.0 | 911.1 | 4746.3 | 1157.9 | 6075.0 |
| 14 | 6 | 2320.5 | 1335.1 | 545.1 | 919.4 | 3721.7 | 1189.5 | 4075.0 |
| C | 4 | 1485.4 | 988.0 | 494.0 | -86.8 | 3057.6 | 873.7 | 2956.8 |
| Total | 28 | 4196.1 | 2951.4 | 557.8 | 3051.7 | 5340.6 | 873.7 | 9512.8 |

C: control

| **Independent-Samples Kruskal-Wallis Test Summary** | |
| --- | --- |
| Total N | 28 |
| Test Statistic | 19.950^a^ |
| Degree Of Freedom | 5 |
| Asymptotic Sig.(2-sided test) | 0.001 |
| a. The test statistic is adjusted for ties. | |

| **Pairwise Comparisons of Recovery day** | | | | | |
| --- | --- | --- | --- | --- | --- |
| Sample 1-Sample 2 | Test Statistic | Std. Error | Std. Test Statistic | Sig. | Adj. Sig.^a^ |
| C-Day 14 | -5.417 | 5.309 | -1.020 | 0.308 | 1.000 |
| C-Day 7 | -6.583 | 5.309 | -1.240 | 0.215 | 1.000 |
| C-Day 3 | -11.000 | 5.816 | -1.891 | 0.059 | 1.000 |
| C-Day 0 | -18.500 | 5.816 | -3.181 | 0.001 | 0.031 |
| C-Day 1 | -20.750 | 5.816 | -3.568 | <0.001 | 0.008 |
| Day 14-Day 7 | -1.167 | 4.749 | -0.246 | 0.806 | 1.000 |
| Day 14-Day 3 | -5.583 | 5.309 | -1.052 | 0.293 | 1.000 |
| Day 14-Day 0 | 13.083 | 5.309 | 2.464 | 0.014 | 0.288 |
| Day 14-Day 1 | 15.333 | 5.309 | 2.888 | 0.004 | 0.081 |
| Day 7-Day 3 | 4.417 | 5.309 | 0.832 | 0.405 | 1.000 |
| Day 7-Day 0 | 11.917 | 5.309 | 2.245 | 0.025 | 0.521 |
| Day 7-Day 1 | 14.167 | 5.309 | 2.668 | 0.008 | 0.160 |
| Day 3-Day 0 | 7.500 | 5.816 | 1.290 | 0.197 | 1.000 |
| Day 3-Day 1 | 9.750 | 5.816 | 1.676 | 0.094 | 1.000 |
| Day 0-Day 1 | -2.250 | 5.816 | -0.387 | 0.699 | 1.000 |
| Each row tests the null hypothesis that the Sample 1 and Sample 2 distributions are the same.  Asymptotic significances (2-sided tests) are displayed. The significance level is 0.05. | | | | | |
| a. Significance values have been adjusted by the Bonferroni correction for multiple tests. | | | | | |

C=control

**Fig 3. ELISA for inflammatory cytokines and growth factors in the lung tissue, (C) TNF-α**

| **Tests of Normality** | | | | | | |
| --- | --- | --- | --- | --- | --- | --- |
|  | Kolmogorov-Smirnov^a^ | | | Shapiro-Wilk | | |
|  | Statistic | df | Sig. | Statistic | df | Sig. |
| TNF-a pg/g | 0.131 | 28 | 0.200^*^ | 0.937 | 28 | 0.093 |
| *. This is a lower bound of the true significance. | | | | | | |
| a. Lilliefors Significance Correction | | | | | | |

| **Descriptives** | | | | | | | | |
| --- | --- | --- | --- | --- | --- | --- | --- | --- |
| TNF-a pg/g | | | | | | | | |
| Day after VILI | N | Mean | Std. Deviation | Std. Error | 95% Confidence Interval for Mean | | Minimum | Maximum |
|  |  |  |  |  | Lower Bound | Upper Bound |  |  |
| 0 | 4 | 39676.1 | 1900.8 | 950.4 | 36651.5 | 42700.7 | 37500.0 | 41554.9 |
| 1 | 4 | 52728.1 | 4400.4 | 2200.2 | 45726.2 | 59730.1 | 47700.0 | 56812.5 |
| 3 | 4 | 35226.7 | 3372.1 | 1686.1 | 29860.9 | 40592.5 | 32100.0 | 38769.5 |
| 7 | 6 | 27948.4 | 4043.6 | 1650.8 | 23704.9 | 32191.8 | 22500.0 | 32169.3 |
| 14 | 6 | 23359.9 | 4811.1 | 1964.1 | 18311.0 | 28408.9 | 16500.0 | 28051.7 |
| C | 4 | 21293.8 | 2792.6 | 1396.3 | 16850.1 | 25737.4 | 19000.0 | 25200.0 |
| Total | 28 | 32269.6 | 11093.4 | 2096.5 | 27968.0 | 36571.2 | 16500.0 | 56812.5 |

C: control

| **Test of Homogeneity of Variances** | | | |
| --- | --- | --- | --- |
| TNF-a pg/g | | | |
| Levene Statistic | df1 | df2 | Sig. |
| 2.420 | 5 | 22 | 0.068 |

| **ANOVA** | | | | | |
| --- | --- | --- | --- | --- | --- |
| TNF-a pg/g | | | | | |
|  | Sum of Squares | df | Mean Square | F | Sig. |
| Between Groups | 2998811450.391 | 5 | 599762290.078 | 40.734 | <0.001 |
| Within Groups | 323923975.065 | 22 | 14723817.048 |  |  |
| Total | 3322735425.457 | 27 |  |  |  |

| **Multiple Comparisons** | | | | | | | | | |
| --- | --- | --- | --- | --- | --- | --- | --- | --- | --- |
| Dependent Variable:TNF-a pg/g | | | | | | | | | |
|  | (I) Day after VILI | | (J) Day after VILI | | Mean Difference (I-J) | Std. Error | Sig. | 95% Confidence Interval | |
|  |  |  |  |  |  |  |  | Lower Bound | Upper Bound |
| Bonferroni |  | 0 |  | 1 | -13052.0675 | 2713.2837 | 0.001 | -21981.179 | -4122.956 |
|  |  |  |  | 3 | 4449.3600 | 2713.2837 | 1.000 | -4479.752 | 13378.472 |
|  |  |  |  | 7 | 11727.6808 | 2476.8778 | 0.002 | 3576.555 | 19878.807 |
|  |  |  |  | 14 | 16316.1092 | 2476.8778 | <0.001 | 8164.983 | 24467.235 |
|  |  |  |  | C | 18382.3075 | 2713.2837 | <0.001 | 9453.196 | 27311.419 |
|  |  | 1 |  | 0 | 13052.0675 | 2713.2837 | 0.001 | 4122.956 | 21981.179 |
|  |  |  |  | 3 | 17501.4275 | 2713.2837 | <0.001 | 8572.316 | 26430.539 |
|  |  |  |  | 7 | 24779.7483 | 2476.8778 | <0.001 | 16628.622 | 32930.875 |
|  |  |  |  | 14 | 29368.1767 | 2476.8778 | <0.001 | 21217.050 | 37519.303 |
|  |  |  |  | C | 31434.3750 | 2713.2837 | <0.001 | 22505.263 | 40363.487 |
|  |  | 3 |  | 0 | -4449.3600 | 2713.2837 | 1.000 | -13378.472 | 4479.752 |
|  |  |  |  | 1 | -17501.4275 | 2713.2837 | <0.001 | -26430.539 | -8572.316 |
|  |  |  |  | 7 | 7278.3208 | 2476.8778 | 0.114 | -872.805 | 15429.447 |
|  |  |  |  | 14 | 11866.7492 | 2476.8778 | 0.001 | 3715.623 | 20017.875 |
|  |  |  |  | C | 13932.9475 | 2713.2837 | 0.001 | 5003.836 | 22862.059 |
|  |  | 7 |  | 0 | -11727.6808 | 2476.8778 | 0.002 | -19878.807 | -3576.555 |
|  |  |  |  | 1 | -24779.7483 | 2476.8778 | <0.001 | -32930.875 | -16628.622 |
|  |  |  |  | 3 | -7278.3208 | 2476.8778 | 0.114 | -15429.447 | 872.805 |
|  |  |  |  | 14 | 4588.4283 | 2215.3869 | 0.754 | -2702.161 | 11879.017 |
|  |  |  |  | C | 6654.6267 | 2476.8778 | 0.202 | -1496.500 | 14805.753 |
|  |  | 14 |  | 0 | -16316.1092 | 2476.8778 | <0.001 | -24467.235 | -8164.983 |
|  |  |  |  | 1 | -29368.1767 | 2476.8778 | <0.001 | -37519.303 | -21217.050 |
|  |  |  |  | 3 | -11866.7492 | 2476.8778 | 0.001 | -20017.875 | -3715.623 |
|  |  |  |  | 7 | -4588.4283 | 2215.3869 | 0.754 | -11879.017 | 2702.161 |
|  |  |  |  | C | 2066.1983 | 2476.8778 | 1.000 | -6084.928 | 10217.325 |
|  |  | C |  | 0 | -18382.3075 | 2713.2837 | <0.001 | -27311.419 | -9453.196 |
|  |  |  |  | 1 | -31434.3750 | 2713.2837 | <0.001 | -40363.487 | -22505.263 |
|  |  |  |  | 3 | -13932.9475 | 2713.2837 | 0.001 | -22862.059 | -5003.836 |
|  |  |  |  | 7 | -6654.6267 | 2476.8778 | 0.202 | -14805.753 | 1496.500 |
|  |  |  |  | 14 | -2066.1983 | 2476.8778 | 1.000 | -10217.325 | 6084.928 |

C: control

**Fig 3. ELISA for inflammatory cytokines and growth factors in the lung tissue, (D) TGF-β**

| **Tests of Normality** | | | | | | |
| --- | --- | --- | --- | --- | --- | --- |
|  | Kolmogorov-Smirnov^a^ | | | Shapiro-Wilk | | |
|  | Statistic | df | Sig. | Statistic | df | Sig. |
| TGF-b pg/dL | 0.192 | 28 | 0.009 | 0.872 | 28 | 0.003 |
| a. Lilliefors Significance Correction | | | | | | |

| **Descriptives** | | | | | | | | |
| --- | --- | --- | --- | --- | --- | --- | --- | --- |
| TGF-b pg/g | | | | | | | | |
| Day after VILI | N | Mean | Std. Deviation | Std. Error | 95% Confidence Interval for Mean | | Minimum | Maximum |
|  |  |  |  |  | Lower Bound | Upper Bound |  |  |
| 0 | 4 | 3032.0 | 184.5 | 92.2 | 2738.4 | 3325.6 | 2848.0 | 3252.0 |
| 1 | 4 | 1630.5 | 1211.3 | 605.7 | -297.0 | 3558.0 | 744.0 | 3344.0 |
| 3 | 4 | 7215.0 | 178.7 | 89.3 | 6930.7 | 7499.3 | 7000.0 | 7424.0 |
| 7 | 6 | 9902.7 | 1146.4 | 468.0 | 8699.6 | 11105.8 | 8718.0 | 11304.0 |
| 14 | 6 | 11542.3 | 117.2 | 47.8 | 11419.3 | 11665.3 | 11408.0 | 11730.0 |
| C | 4 | 2895.6 | 1146.3 | 573.1 | 1071.6 | 4719.6 | 2194.0 | 4608.4 |
| Total | 28 | 6705.8 | 4004.5 | 756.8 | 5153.0 | 8258.6 | 744.0 | 11730.0 |

C: control

| **Independent-Samples Kruskal-Wallis Test Summary** | |
| --- | --- |
| Total N | 28 |
| Test Statistic | 24.835^a^ |
| Degree Of Freedom | 5 |
| Asymptotic Sig.(2-sided test) | <0.001 |
| a. The test statistic is adjusted for ties. | |

| **Pairwise Comparisons of Recovery day** | | | | | |
| --- | --- | --- | --- | --- | --- |
| Sample 1-Sample 2 | Test Statistic | Std. Error | Std. Test Statistic | Sig. | Adj. Sig.^a^ |
| Day 1-C | 2.500 | 5.817 | 0.430 | 0.667 | 1.000 |
| Day 1-Day 0 | 4.250 | 5.817 | 0.731 | 0.465 | 1.000 |
| Day 1-Day 3 | -10.250 | 5.817 | -1.762 | 0.078 | 1.000 |
| Day 1-Day 7 | -15.250 | 5.310 | -2.872 | 0.004 | 0.086 |
| Day 1-Day 14 | -21.250 | 5.310 | -4.002 | <0.001 | 0.001 |
| C-Day 0 | -1.750 | 5.817 | -0.301 | 0.764 | 1.000 |
| C-Day 3 | -7.750 | 5.817 | -1.332 | 0.183 | 1.000 |
| C-Day 7 | -12.750 | 5.310 | -2.401 | 0.016 | 0.343 |
| C-Day 14 | -18.750 | 5.310 | -3.531 | <0.001 | 0.009 |
| Day 0-Day 3 | -6.000 | 5.817 | -1.032 | 0.302 | 1.000 |
| Day 0-Day 7 | -11.000 | 5.310 | -2.072 | 0.038 | 0.804 |
| Day 0-Day 14 | -17.000 | 5.310 | -3.202 | 0.001 | 0.029 |
| Day 3-Day 7 | -5.000 | 5.310 | -0.942 | 0.346 | 1.000 |
| Day 3-Day 14 | 11.000 | 5.310 | 2.072 | 0.038 | 0.804 |
| Day 7-Day 14 | 6.000 | 4.749 | 1.263 | 0.206 | 1.000 |
| Each row tests the null hypothesis that the Sample 1 and Sample 2 distributions are the same.  Asymptotic significances (2-sided tests) are displayed. The significance level is 0.05. | | | | | |
| a. Significance values have been adjusted by the Bonferroni correction for multiple tests. | | | | | |

C= control

**Fig 3. ELISA for inflammatory cytokines and growth factors in the lung tissue, (E) VEGF**

| **Tests of Normality** | | | | | | |
| --- | --- | --- | --- | --- | --- | --- |
|  | Kolmogorov-Smirnov^a^ | | | Shapiro-Wilk | | |
|  | Statistic | df | Sig. | Statistic | df | Sig. |
| VEGF pg/g | 0.181 | 28 | 0.019 | 0.873 | 28 | 0.003 |
| a. Lilliefors Significance Correction | | | | | | |

| **Descriptives** | | | | | | | | |
| --- | --- | --- | --- | --- | --- | --- | --- | --- |
| VEGF pg/g | | | | | | | | |
| Day after VILI | N | Mean | Std. Deviation | Std. Error | 95% Confidence Interval for Mean | | Minimum | Maximum |
|  |  |  |  |  | Lower Bound | Upper Bound |  |  |
| 0 | 4 | 6678.8 | 254.6 | 127.3 | 6273.8 | 7083.9 | 6345.0 | 6955.0 |
| 1 | 4 | 5276.6 | 95.2 | 47.6 | 5125.1 | 5428.1 | 5200.0 | 5403.9 |
| 3 | 4 | 10919.4 | 1210.3 | 605.1 | 8993.6 | 12845.2 | 9125.0 | 11727.5 |
| 7 | 6 | 12556.0 | 567.5 | 231.7 | 11960.5 | 13151.5 | 11540.0 | 13195.0 |
| 14 | 6 | 13755.0 | 138.5 | 56.6 | 13609.6 | 13900.4 | 13595.0 | 13935.0 |
| C | 4 | 8190.2 | 505.6 | 252.8 | 7385.7 | 8994.6 | 7513.3 | 8725.0 |
| Total | 28 | 10075.9 | 3223.6 | 609.2 | 8826.0 | 11325.9 | 5200.0 | 13935.0 |

C: control

| **Independent-Samples Kruskal-Wallis Test Summary** | |
| --- | --- |
| Total N | 28 |
| Test Statistic | 25.916^a^ |
| Degree Of Freedom | 5 |
| Asymptotic Sig.(2-sided test) | <0.001 |
| a. The test statistic is adjusted for ties. | |

| **Pairwise Comparisons of Recovery day** | | | | | |
| --- | --- | --- | --- | --- | --- |
| Sample 1-Sample 2 | Test Statistic | Std. Error | Std. Test Statistic | Sig. | Adj. Sig.^a^ |
| Day 1-Day 0 | 4.000 | 5.817 | 0.688 | 0.492 | 1.000 |
| Day 1-C | 8.000 | 5.817 | 1.375 | 0.169 | 1.000 |
| Day 1-Day 3 | -12.500 | 5.817 | -2.149 | 0.032 | 0.664 |
| Day 1-Day 7 | -16.667 | 5.310 | -3.139 | 0.002 | 0.036 |
| Day 1-Day 14 | -23.000 | 5.310 | -4.332 | <0.001 | <0.001 |
| Day 0-C | 4.000 | 5.817 | 0.688 | 0.492 | 1.000 |
| Day 0-Day 3 | -8.500 | 5.817 | -1.461 | 0.144 | 1.000 |
| Day 0-Day 7 | -12.667 | 5.310 | -2.386 | 0.017 | 0.358 |
| Day 0-Day 14 | -19.000 | 5.310 | -3.578 | <0.001 | 0.007 |
| C-Day 3 | -4.500 | 5.817 | -0.774 | 0.439 | 1.000 |
| C-Day 7 | -8.667 | 5.310 | -1.632 | 0.103 | 1.000 |
| C-Day 14 | -15.000 | 5.310 | -2.825 | 0.005 | 0.099 |
| Day 3-Day 7 | -4.167 | 5.310 | -0.785 | 0.433 | 1.000 |
| Day 3-Day 14 | 10.500 | 5.310 | 1.977 | 0.048 | 1.000 |
| Day 7-Day 14 | 6.333 | 4.749 | 1.334 | 0.182 | 1.000 |
| Each row tests the null hypothesis that the Sample 1 and Sample 2 distributions are the same.  Asymptotic significances (2-sided tests) are displayed. The significance level is 0.05. | | | | | |
| a. Significance values have been adjusted by the Bonferroni correction for multiple tests. | | | | | |

C= control

**Fig 4. (C-D) Histometric analysis of the time courses of VEGF expression and the number of Ki67-positive cells in the lung alveolar epithelium**

| **Tests of Normality** | | | | | | | | | | | | |
| --- | --- | --- | --- | --- | --- | --- | --- | --- | --- | --- | --- | --- |
|  | Kolmogorov-Smirnov | | | | | | Shapiro-Wilk | | | | | |
|  | Statistic | | df | | Sig. | | Statistic | | df | | Sig. | |
| VEGF relative staining intensity | 0.337 | | 36 | | <0.001 | | 0.723 | | 36 | | <0.001 | |
|  | | | | | | | | | | | | |
| **Tests of Normality** | | | | | | | | | | | | |
|  | | Kolmogorov-Smirnov | | | | | | Shapiro-Wilk | | | | |
|  |  | Statistic | | df | | Sig. | | Statistic | | df | | Sig. |
| Ki67 positive cell/100 alveoli | | 0.239 | | 36 | | <0.001 | | 0.797 | | 36 | | <0.001 |
|  | | | | | | | | | | | | |

| **Descriptives** | | | | | | | | | |
| --- | --- | --- | --- | --- | --- | --- | --- | --- | --- |
|  | Day after VILI | N | Mean | Std. Deviation | Std. Error | 95% Confidence Interval for Mean | | Minimum | Maximum |
|  |  |  |  |  |  | Lower Bound | Upper Bound |  |  |
| VEGF relative staining intensity | 0 | 6 | 102.8 | 6.7 | 2.7 | 95.8 | 109.9 | 96.0 | 112.0 |
|  | 1 | 6 | 77.3 | 7.3 | 3.0 | 69.6 | 85.0 | 71.0 | 89.0 |
|  | 3 | 6 | 213.7 | 35.2 | 14.4 | 176.7 | 250.6 | 156.0 | 263.0 |
|  | 7 | 6 | 991.8 | 119.9 | 49.0 | 866.0 | 1117.7 | 837.0 | 1150.0 |
|  | 14 | 6 | 2165.8 | 245.5 | 100.2 | 1908.2 | 2423.5 | 1854.0 | 2489.0 |
|  | C | 6 | 252.8 | 25.9 | 10.6 | 225.7 | 280.0 | 221.0 | 294.0 |
|  | Total | 36 | 634.1 | 769.3 | 128.2 | 373.8 | 894.4 | 71.0 | 2489.0 |
| Ki67 positive cell/100 alveoli | 0 | 6 | 53 | 7 | 3 | 46 | 60 | 43 | 59 |
|  | 1 | 6 | 48 | 5 | 2 | 43 | 53 | 41 | 54 |
|  | 3 | 6 | 84 | 5 | 2 | 78 | 89 | 76 | 89 |
|  | 7 | 6 | 106 | 4 | 2 | 102 | 111 | 101 | 113 |
|  | 14 | 6 | 216 | 36 | 15 | 178 | 254 | 183 | 271 |
|  | C | 6 | 87 | 5 | 2 | 81 | 93 | 81 | 95 |
|  | Total | 36 | 99 | 59 | 10 | 79 | 119 | 41 | 271 |

C: control

**VEGF relative staining intensity**

| **Independent-Samples Kruskal-Wallis Test Summary** | |
| --- | --- |
| Total N | 36 |
| Test Statistic | 33.563^a^ |
| Degree Of Freedom | 5 |
| Asymptotic Sig.(2-sided test) | <0.001 |
| a. The test statistic is adjusted for ties. | |

| **Pairwise Comparisons of Days after VILI** | | | | | |
| --- | --- | --- | --- | --- | --- |
| Sample 1-Sample 2 | Test Statistic | Std. Error | Std. Test Statistic | Sig. | Adj. Sig.^a^ |
| Day 1-Day 0 | 6.000 | 6.082 | 0.987 | 0.324 | 1.000 |
| Day 1-Day 3 | -12.917 | 6.082 | -2.124 | 0.034 | 0.505 |
| Day 1-C | 17.083 | 6.082 | 2.809 | 0.005 | 0.075 |
| Day 1-Day 7 | -24.000 | 6.082 | -3.946 | <0.001 | 0.001 |
| Day 1-Day 14 | -30.000 | 6.082 | -4.933 | <0.001 | <0.001 |
| Day 0-Day 3 | -6.917 | 6.082 | -1.137 | 0.255 | 1.000 |
| Day 0-C | 11.083 | 6.082 | 1.822 | 0.068 | 1.000 |
| Day 0-Day 7 | -18.000 | 6.082 | -2.960 | 0.003 | 0.046 |
| Day 0-Day 14 | -24.000 | 6.082 | -3.946 | <0.001 | 0.001 |
| Day 3-C | 4.167 | 6.082 | 0.685 | 0.493 | 1.000 |
| Day 3-Day 7 | -11.083 | 6.082 | -1.822 | 0.068 | 1.000 |
| Day 3-Day 14 | 17.083 | 6.082 | 2.809 | 0.005 | 0.075 |
| C-Day 7 | -6.917 | 6.082 | -1.137 | 0.255 | 1.000 |
| C-Day 14 | -12.917 | 6.082 | -2.124 | 0.034 | 0.505 |
| Day 7-Day 14 | 6.000 | 6.082 | 0.987 | 0.324 | 1.000 |
| Each row tests the null hypothesis that the Sample 1 and Sample 2 distributions are the same.  Asymptotic significances (2-sided tests) are displayed. The significance level is 0.05. | | | | | |
| a. Significance values have been adjusted by the Bonferroni correction for multiple tests. | | | | | |

C=control

**Ki67 positive cell/100 alveoli**

| **Independent-Samples Kruskal-Wallis Test Summary** | |
| --- | --- |
| Total N | 36 |
| Test Statistic | 32.428^a^ |
| Degree Of Freedom | 5 |
| Asymptotic Sig.(2-sided test) | <0.001 |
| a. The test statistic is adjusted for ties. | |

| **Pairwise Comparisons of Days after VILI** | | | | | |
| --- | --- | --- | --- | --- | --- |
| Sample 1-Sample 2 | Test Statistic | Std. Error | Std. Test Statistic | Sig. | Adj. Sig.^a^ |
| Day 1-Day 0 | 2.833 | 6.082 | 0.466 | 0.641 | 1.000 |
| Day 1-Day 3 | -12.500 | 6.082 | -2.055 | 0.040 | 0.598 |
| Day 1-C | 14.333 | 6.082 | 2.357 | 0.018 | 0.276 |
| Day 1-Day 7 | -22.417 | 6.082 | -3.686 | <0.001 | 0.003 |
| Day 1-Day 14 | -28.417 | 6.082 | -4.673 | <0.001 | <0.001 |
| Day 0-Day 3 | -9.667 | 6.082 | -1.589 | 0.112 | 1.000 |
| Day 0-C | 11.500 | 6.082 | 1.891 | 0.059 | 0.879 |
| Day 0-Day 7 | -19.583 | 6.082 | -3.220 | 0.001 | 0.019 |
| Day 0-Day 14 | -25.583 | 6.082 | -4.207 | <0.001 | <0.001 |
| Day 3-C | 1.833 | 6.082 | 0.301 | 0.763 | 1.000 |
| Day 3-Day 7 | -9.917 | 6.082 | -1.631 | 0.103 | 1.000 |
| Day 3-Day 14 | 15.917 | 6.082 | 2.617 | 0.009 | 0.133 |
| C-Day 7 | -8.083 | 6.082 | -1.329 | 0.184 | 1.000 |
| C-Day 14 | -14.083 | 6.082 | -2.316 | 0.021 | 0.309 |
| Day 7-Day 14 | 6.000 | 6.082 | 0.987 | 0.324 | 1.000 |
| Each row tests the null hypothesis that the Sample 1 and Sample 2 distributions are the same.  Asymptotic significances (2-sided tests) are displayed. The significance level is 0.05. | | | | | |
| a. Significance values have been adjusted by the Bonferroni correction for multiple tests.  C= control | | | | | |

**Fig 4. (E) Correlation between the VEGF relative staining intensity and** **the number of Ki67-positive cells per 100 alveoli**

| **Correlations** | | | | |
| --- | --- | --- | --- | --- |
|  | | | VEGF relative staining intensity | Ki67 positive cell/100 alveoli |
| Spearman's rho | VEGF relative staining intensity | Correlation Coefficient | 1.000 | 0.926^**^ |
|  |  | Sig. (2-tailed) | . | <0.001 |
|  |  | N | 36 | 36 |
|  | Ki67 positive cell/100 alveoli | Correlation Coefficient | 0.926^**^ | 1.000 |
|  |  | Sig. (2-tailed) | <0.001 | . |
|  |  | N | 36 | 36 |
| **. Correlation is significant at the 0.01 level (2-tailed). | | | | |

**Fig 5. Time course of VEGF mRNA expression in lung-recruited monocytes following VILI.**

| **Tests of Normality** | | | | | | |
| --- | --- | --- | --- | --- | --- | --- |
|  | Kolmogorov-Smirnov^a^ | | | Shapiro-Wilk | | |
|  | Statistic | df | Sig. | Statistic | df | Sig. |
| VEGF mRNA | 0.135 | 26 | 0.200^*^ | 0.934 | 26 | 0.096 |
| *. This is a lower bound of the true significance. | | | | | | |
| a. Lilliefors Significance Correction | | | | | | |

| **Descriptives** | | | | | | | | |
| --- | --- | --- | --- | --- | --- | --- | --- | --- |
| VEGF mRNA | | | | | | | | |
| Day after VILI | N | Mean | Std. Deviation | Std. Error | 95% Confidence Interval for Mean | | Minimum | Maximum |
|  |  |  |  |  | Lower Bound | Upper Bound |  |  |
| 0 | 4 | 0.19 | 0.05 | 0.02 | 0.11 | 0.27 | 0.14 | 0.25 |
| 1 | 4 | 0.04 | 0.02 | 0.01 | 0.01 | 0.07 | 0.02 | 0.05 |
| 3 | 4 | 0.52 | 0.08 | 0.04 | 0.39 | 0.64 | 0.41 | 0.60 |
| 7 | 5 | 0.68 | 0.22 | 0.10 | 0.41 | 0.96 | 0.43 | 0.94 |
| 14 | 5 | 1.17 | 0.22 | 0.10 | 0.89 | 1.45 | 0.92 | 1.39 |
| C | 4 | 1.01 | 0.17 | 0.08 | 0.74 | 1.27 | 0.89 | 1.26 |
| Total | 26 | 0.63 | 0.44 | 0.09 | 0.45 | 0.80 | 0.02 | 1.39 |

C: control

| **Test of Homogeneity of Variances** | | | |
| --- | --- | --- | --- |
| VEGF mRNA | | | |
| Levene Statistic | df1 | df2 | Sig. |
| 5.232 | 5 | 20 | 0.003 |

| **ANOVA** | | | | | |
| --- | --- | --- | --- | --- | --- |
| VEGF mRNA | | | | | |
|  | Sum of Squares | df | Mean Square | F | Sig. |
| Between Groups | 4.243 | 5 | 0.849 | 33.717 | <0.001 |
| Within Groups | 0.50 | 20 | 0.025 |  |  |
| Total | 4.746 | 25 |  |  |  |

| **Multiple Comparisons** | | | | | | | | | |
| --- | --- | --- | --- | --- | --- | --- | --- | --- | --- |
| Dependent Variable: VEGF mRNA | | | | | | | | | |
|  | (I) day after VILI | | (J) day after VILI | | Mean Difference (I-J) | Std. Error | Sig. | 95% Confidence Interval | |
|  |  |  |  |  |  |  |  | Lower Bound | Upper Bound |
| Dunnett T3 |  | 0 |  | 1 | 0.14974 | 0.02547 | 0.036 | 0.0142 | 0.2853 |
|  |  |  |  | 3 | -0.32462 | 0.04578 | 0.008 | -0.5362 | -0.1130 |
|  |  |  |  | 7 | -0.49370 | 0.10103 | 0.049 | -0.9854 | -0.0020 |
|  |  |  |  | 14 | -0.97617 | 0.10321 | 0.004 | -1.4797 | -0.4726 |
|  |  |  |  | C | -0.81842 | 0.08650 | 0.009 | -1.3041 | -0.3327 |
|  |  | 1 |  | 0 | -0.14974 | 0.02547 | 0.036 | -0.2853 | -0.0142 |
|  |  |  |  | 3 | -0.47436 | 0.04003 | 0.005 | -0.7076 | -0.2411 |
|  |  |  |  | 7 | -0.64344 | 0.09855 | 0.021 | -1.1480 | -0.1389 |
|  |  |  |  | 14 | -1.12591 | 0.10080 | 0.003 | -1.6422 | -0.6097 |
|  |  |  |  | C | -0.96816 | 0.08360 | 0.008 | -1.4826 | -0.4538 |
|  |  | 3 |  | 0 | 0.32462 | 0.04578 | 0.008 | 0.1130 | 0.5362 |
|  |  |  |  | 1 | 0.47436 | 0.04003 | 0.005 | 0.2411 | 0.7076 |
|  |  |  |  | 7 | -0.16908 | 0.10564 | 0.779 | -0.6480 | 0.3098 |
|  |  |  |  | 14 | -0.65155 | 0.10773 | 0.015 | -1.1420 | -0.1611 |
|  |  |  |  | C | -0.49380 | 0.09185 | 0.038 | -0.9519 | -0.0356 |
|  |  | 7 |  | 0 | 0.49370 | 0.10103 | 0.049 | 0.0020 | 0.9854 |
|  |  |  |  | 1 | 0.64344 | 0.09855 | 0.021 | 0.1389 | 1.1480 |
|  |  |  |  | 3 | 0.16908 | 0.10564 | 0.779 | -0.3098 | 0.6480 |
|  |  |  |  | 14 | -0.48247 | 0.14042 | 0.092 | -1.0289 | 0.0639 |
|  |  |  |  | C | -0.32472 | 0.12863 | 0.320 | -0.8459 | 0.1965 |
|  |  | 14 |  | 0 | 0.97617 | 0.10321 | 0.004 | 0.4726 | 1.4797 |
|  |  |  |  | 1 | 1.12591 | 0.10080 | 0.003 | 0.6097 | 1.6422 |
|  |  |  |  | 3 | 0.65155 | 0.10773 | 0.015 | 0.1611 | 1.1420 |
|  |  |  |  | 7 | 0.48247 | 0.14042 | 0.092 | -0.0639 | 1.0289 |
|  |  |  |  | C | 0.15775 | 0.13036 | 0.945 | -0.3707 | 0.6862 |
|  |  | C |  | 0 | 0.81842 | 0.08650 | 0.009 | 0.3327 | 1.3041 |
|  |  |  |  | 1 | 0.96816 | 0.08360 | 0.008 | 0.4538 | 1.4826 |
|  |  |  |  | 3 | 0.49380 | 0.09185 | 0.038 | 0.0356 | 0.9519 |
|  |  |  |  | 7 | 0.32472 | 0.12863 | 0.320 | -0.1965 | 0.8459 |
|  |  |  |  | 14 | -0.15775 | 0.13036 | 0.945 | -0.6862 | 0.3707 |

C: control

**Fig 6. Pulmonary VEGF protein levels in mice without and with depletion of monocytes on days 7 and 14 after VILI.**

**Day 7 after VILI**

| **Group Statistics** | | | | | | |
| --- | --- | --- | --- | --- | --- | --- |
|  | Day after VILI | depletion protocol | N | Mean | Std. Deviation | Std. Error Mean |
| VEGF | 7 | without depletion | 6 | 12556.0 | 567.5 | 231.7 |
|  | 7 | depletion on day 3 | 6 | 9544.4 | 1069.9 | 436.8 |

**Mann-Whitney U Test**

| **Ranks** | | | | |
| --- | --- | --- | --- | --- |
|  | depletion protocol | N | Mean Rank | Sum of Ranks |
| VEGF | without depletion | 6 | 9.50 | 57.00 |
|  | depletion on day 3 | 6 | 3.50 | 21.00 |
|  | Total | 12 |  |  |

| **Test Statistics^a^** | |
| --- | --- |
|  | VEGF |
| Mann-Whitney U | <0.001 |
| Wilcoxon W | 21.000 |
| Z | -2.882 |
| Asymp. Sig. (2-tailed) | 0.004 |
| Exact Sig. [2*(1-tailed Sig.)] | 0.002^b^ |
| a. Grouping Variable: depletion protocol | |
| b. Not corrected for ties. | |

**Day 14 after VILI**

| **Group Statistics** | | | | | | |
| --- | --- | --- | --- | --- | --- | --- |
|  | day after VILI | depletion protocol | N | Mean | Std. Deviation | Std. Error Mean |
| VEGF | 14 | without depletion | 6 | 13755.0 | 138.5 | 56.6 |
|  | 14 | depletion on day 3,7,11 | 6 | 7301.9 | 1602.8 | 654.3 |

**Mann-Whitney U Test**

| **Ranks** | | | | |
| --- | --- | --- | --- | --- |
|  | depletion protocol | N | Mean Rank | Sum of Ranks |
| VEGF | without depletion | 6 | 9.50 | 57.00 |
|  | depletion on day 3,7,11 | 6 | 3.50 | 21.00 |
|  | Total | 12 |  |  |

| **Test Statistics^a^** | |
| --- | --- |
|  | VEGF |
| Mann-Whitney U | <0.001 |
| Wilcoxon W | 21.000 |
| Z | -2.882 |
| Asymp. Sig. (2-tailed) | 0.004 |
| Exact Sig. [2*(1-tailed Sig.)] | 0.002^b^ |
| a. Grouping Variable: depletion protocol | |
| b. Not corrected for ties. | |
