# Supplementary material for: Roles of lung-recruited monocytes and pulmonary Vascular Endothelial Growth Factor (VEGF) in resolving Ventilator-Induced Lung Injury (VILI)
Source: PLoS One. 2021 Mar 19;16(3):e0248959. doi: 10.1371/journal.pone.0248959 (PMC7978382; doi:10.1371/journal.pone.0248959)
Supplement: Attachment — Submitted filename: Response to reviewers.docx [file pone.0248959.s003.docx]

Chung-Sheng Shi

Chang Gung University

Graduate Institute of Clinical Medicine Sciences

No. 259, Wenhua 1st Rd

Guishan Dist., Taoyuan City 33302, Taiwan

csshi@mail.cgu.edu.tw

Dr. Heinz Fehrenbach

Academic Editor

*PLOS ONE*

November 18, 2020

Subject: Revision and resubmission of manuscript PONE-D-20-27843

Dear Dr. Heinz Fehrenbach,

Thank you for your letter and the opportunity to revise our paper in *PLOS ONE*. We appreciate your insightful comments on revising the Materials & Methods of the paper.

I have included the comments immediately after this letter and responded to them individually, indicating exactly how we addressed each concern or problem and describing the changes we have made. The revisions have been approved by all four authors, and I have again been chosen as the corresponding author. The changes are marked in red in the paper as you requested, and the marked and unmarked versions of the revised manuscript have been uploaded to the online submission system.

In response to your comments on the Materials & Methods section, we have described the number of animals per group for each experiment and the number of replications in each experiment. In addition, we have included in the main text detailed descriptions of the methods, which were originally presented in the supplementary information.

We hope the revised manuscript will better suit *PLOS ONE* but are happy to consider further revisions, and we thank you for your continued interest in our research.

Sincerely,

Chung-Sheng Shi

Graduate Institute of Clinical Medicine Sciences, College of Medicine, Chang Gung University,

**Editor comments,** **Journal Requirements, Author Responses and Manuscript Changes**

Editor Comments:
Before inviting expert reviewers, I checked your manuscript and noticed that there are neither details about the number of animals per group and experiments nor about the number of relications of experiments. Please include these data in the Materials & Methods section as well as in the figure legends. In addition, you may wish to include detailed descriptions of the methods, which you give in the supplement, directly into the main text.
Response:

We have rewritten and detailed the number of animals per group in each experiment and the number of replications for each experiment in the Materials and Methods section. In addition, we have moved the detailed descriptions of the methods initially presented in the supplementary information directly into the Materials and Methods section of the main text.

Journal Requirements

1. Please ensure that your manuscript meets PLOS ONE's style requirements, including those for file naming. The PLOS ONE style templates can be found at https://journals.plos.org/plosone/s/file?id=wjVg/PLOSOne_formatting_sample_main_body.pdf and https://journals.plos.org/plosone/s/file?id=ba62/PLOSOne_formatting_sample_title_authors_affiliations.pdf.

Response:

We have reformatted our manuscript and file names according to the journal style requirements.

2. We suggest you thoroughly copyedit your manuscript for language usage, spelling, and grammar. If you do not know anyone who can help you do this, you may wish to consider employing a professional scientific editing service. While you may use any professional scientific editing service of your choice, PLOS has partnered with both American Journal Experts (AJE) and Editage to provide discounted services to PLOS authors. Both organizations have experience helping authors meet PLOS guidelines and can provide language editing, translation, manuscript formatting, and figure formatting to ensure that your manuscript meets our submission guidelines. To take advantage of our partnership with AJE, visit the AJE website ([http://learn.aje.com/plos/](about:blank)) for a 15% discount off AJE services. To take advantage of our partnership with Editage, visit the Editage website (www.editage.com) and enter the referral code PLOSEDIT for a 15% discount off Editage services.  If the PLOS editorial team finds any language issues in text that either AJE or Editage has edited, the service provider will re-edit the text for free.

Upon resubmission, please provide the following:

- The name of the colleague or the details of the professional service that edited your manuscript
- A copy of your manuscript showing your changes by either highlighting them or using track changes (uploaded as a *supporting information* file)
- A clean copy of the edited manuscript (uploaded as the new *manuscript* file)

Response:

We have had our manuscript professionally edited by Editage.

We have uploaded a copy with red text highlighting the changes and a clean copy as the new manuscript file.

3. Please provide the specific sequences of the primers used in the PCR analysis.

Response:

The primer sequences used for RT-PCR analysis of VEGF mRNA have been included in the Materials and Methods section (**page 12, line 201**). The primer sequences of VEGF and GADPH (endogenous control) are as follows:

VEGF forward: 5’-ATCATGCGGATCAAACCTCACCA-3’;

VEGF reverse: 5’-TCACCGCCTTGGCTTGTCACAT-3’;

GAPDH forward 5’-CTCACTGGCATGGCCTTCCGTGT-3’,

GAPDH reverse: 5’-GATGTCATCATATTTGGCAGGT-3’.

4. At this time, we ask that you please provide scale bars on the microscopy images presented in Figures 1 and 4, and refer to the scale bar in the corresponding Figure legend.

Response:

We have added scale bars to the microscopy images in Figures 1 and 4 (**page 15, line 251 and page 19, line 342**). The scale bars represent 200 μm, as described in the corresponding figure legends.

5. Please provide the product numbers and any lot numbers of the antibodies purchased for immunohistochemistry in your study.”

Response:

We have edited the Materials and Methods section and detailed the product numbers and lot numbers of the antibodies used for immunohistochemistry analysis in our study (**page 8, line 122**).

6. At this time, we request that you  please report additional details in your Methods section regarding animal care, as per our editorial guidelines:
 
(a) Please state the number of mice used in the study
(b) Please provide details of animal welfare (e.g., shelter, food, water, environmental enrichment);
(c) Please describe the postoperative care received by the animals, including the frequency of monitoring and the criteria used to assess animal health and well-being.

Thank you for your attention to these requests.

Response:

(a) We have described the number of animals per group for each experiment and the number of replications for each experiment in the Materials and Methods section (**page 7, line 112; page 9, line 136; page 10, line 156; page 10, line 167 and page 13, line 211**).
(b) and (c)

We have edited the Methods section and described the animal welfare (**page 6, line 80**) and post-treatment (**page 7, line 107**).

For the animal welfare, all experimental protocols using animals were performed following the Guide to the Care and Use of Experimental Animals and approved by the Institutional Animal Care and Use Committee of Chang Gung Memorial Hospital, Chiayi, Taiwan (Approval number: 2012102301 (September 21, 2013)). All animal experiments were performed in the Animal Center of Chang Gung Memorial Hospital at Chiayi, and mice were housed following IACUC, national, and international animal welfare protocols. The center introduced the AAALAC certification system in 2013 and had obtained AAALAC certification in July 2014 (No: TTQ08384). To optimize environmental conditions for reducing mouse stress on animal welfare, animals were housed in a room with constant temperature (20–25°C) and humidity (40–60%) under a 12‐h light/dark cycle, mouse cages with enrichment toys were limited to 5 mice per cage, animals were given free access to food and water, and the center was maintained in low noises and vibrations.

For the postoperative care, the extubated mice received post-treatment care following the IACUC guidelines. We checked the appearance of mice every day and measured the body weight of mice twice/week. Besides, a full‐time veterinarian would take care of animal health and well-being until they were sacrificed with an overdose of anesthesia on days 1, 3, 7, or 14 after VILI.

7. To comply with PLOS ONE submissions requirements, in your Methods section, please provide additional information regarding the experiments involving animals and ensure you have included details of the type and dosage of anesthesia, both during ventilation and for sacrifice.

Response:

We have detailed the anesthesia of the animals, including the drug, type, and dosage of anesthesia (**page 7, line 97**), during ventilation (**page 7, line 103**), and for sacrifice (**page 7, line 105**). Briefly, mice were anesthetized using intraperitoneal Zoletil 50 (80 mg/kg; Tiletamine-Zolazepam, Virbac, Carros CEDEX, France) and received a single intravenous tail vein injection of 20 ng LPS (O111B4; Sigma-Aldrich, St Louis, MO, USA) immediately before HTV ventilation. The mice were intubated and ventilated with an SAR-830 series small animal ventilator with multi-animal setups (CWE Inc., Ardmore, PA, USA) under room air and general anesthesia with regular intraperitoneal injections of Zoletil 50 (10 mg/kg/h) for 4 h. After ventilation, the mice were sacrificed immediately (day 0) or extubated after spontaneous breath resumption and sacrificed on day 1, 3, 7, or 14 after VILI by an overdose of anesthesia (Zoletil 50, 50 mg/kg).

8. Please ensure that your methods are described in sufficient detail for others to replicate the study. Specifically, please provide the following:
i) Please provide the names of the ELISA kits used to quantify pulmonary growth factors and cytokines.
ii) Please include the Methods described in the appendix of the main manuscript body."

Response:

We have edited the Materials and Methods section and described the names and lot numbers of the ELISA kits used in the quantification of pulmonary growth factors and cytokines (**page 10, line 161**). In addition, we have moved the detailed descriptions of the methods initially presented in the supplement, directly into the Materials and Methods section of the main text.

9. To comply with PLOS ONE submission guidelines, in your Methods section, please provide additional information regarding your statistical analyses. For more information on PLOS ONE's expectations for statistical reporting, please see https://journals.plos.org/plosone/s/submission-guidelines.#loc-statistical-reporting

Response:

We have carefully read PLOS ONE's expectations for statistical reporting and SAMPL guidelines for general guidance on statistical reporting and ensured the description of the statistical analysis section complies with the essential principle. For instance, the name and version of statistical software, the form of summarized data, total sample and group sizes for each analysis, the method used for each analysis, and the alpha level for defining statistical significance has been detailed in the Methods section (**page 13, line 222**). Besides, detailed illustrations of the statistical analysis are provided in the supporting information S1 Text.

10. PLOS requires an ORCID iD for the corresponding author in the Editorial Manager on papers submitted after December 6th, 2016. Please ensure that you have an ORCID iD and that it is validated in the Editorial Manager. To do this, go to update my information (in the upper left-hand corner of the main menu), and click on the Fetch/Validate link next to the ORCID field. This will take you to the ORCID site and allow you to create a new iD or authenticate a pre-existing iD in Editorial Manager. Please see the following video for instructions on linking an ORCID iD to your editorial manager account: https://www.youtube.com/watch?v=_xcclfuvtxQ.

Response:

The ORCID iD of the corresponding author, Chung-Sheng Shi, has been edited, linked, and validated in the online submission system of the Editorial Manager.
