# Supplementary material for: Roles of lung-recruited monocytes and pulmonary Vascular Endothelial Growth Factor (VEGF) in resolving Ventilator-Induced Lung Injury (VILI)
Source: PLoS One. 2021 Mar 19;16(3):e0248959. doi: 10.1371/journal.pone.0248959 (PMC7978382; doi:10.1371/journal.pone.0248959)
Supplement: Attachment — Submitted filename: 20210113 Response to Reviewers.docx [file pone.0248959.s004.docx]

Chung-Sheng Shi

Chang Gung University

Graduate Institute of Clinical Medicine Sciences

No. 259, Wenhua 1st Rd

Guishan Dist., Taoyuan City 33302, Taiwan

csshi@mail.cgu.edu.tw

Dr. Heinz Fehrenbach

Academic Editor

*PLOS ONE*

February 17, 2021

Subject: Revision and resubmission of manuscript PONE-D-20-27843

Dear Dr. Heinz Fehrenbach,

Thank you for your letter and the opportunity to revise our paper in *PLOS ONE* again. In response to the reviewers’ comments, we have detailed point-by-point responses to all comments along with our revised manuscript.

I have included the comments immediately after this letter and responded to them individually, indicating exactly how we addressed each concern or problem and describing the changes we have made. Besides, we have had our manuscript professionally edited by Editage again. All four authors have approved the revisions, and I have again been chosen as the corresponding author. The changes are marked in red in the paper as you requested, and the marked and unmarked versions of the revised manuscript have been uploaded to the online submission system.

We hope the revised manuscript will better suit *PLOS ONE* but are happy to consider further revisions, and we thank you for your continued interest in our research.

Sincerely,

Chung-Sheng Shi

Graduate Institute of Clinical Medicine Sciences, College of Medicine, Chang Gung University,

**Author Responses to Reviewers and Manuscript Changes**

Response to Reviewers

Thank you for your precious comments and advice. Those comments are valuable and helpful for revising and improving our paper and the critical guiding significance to our research. We have studied words carefully and have made corrections, which we hope meet with approval. Revised portions are marked in red on the paper. The major corrections in the paper and the responses to the reviewer’s comments are as following:

Reviewer #1: The resubmitted manuscript entitled „Roles of lung-recruited monocytes and pulmonary vascular endothelial growth factor (VEGF) in resolving ventilator-induced lung injury (VILI)” by Chung-Sheng Shi et al. describes the role of monocytes and the release of VEGF in a model of VILI. The authors described

All points of the reviewer are properly edited the statistics and described the different statistical models in supplement. All numbers of the experiments were mentioned and statistical variances shown as mean ± SD. The text body was well corrected and most of the typos removed. There are some miner points I would like to address.

Response:

Thank you for your summary. We appreciate your efforts in reviewing our manuscript. We have revised the manuscript accordingly. Our point-by-point responses are detailed below:

Minor points:

1. In figure 4B, day 1 the authors should check if the magnification is really the same as in the other histological images. It seems to be a higher magnification. Please clarify.

Response:

We have confirmed that the magnification of the day 1 image in figure 4B is the same as that of other images. The alveoli in this image may be damaged and expanded due to VILI, so it is why the alveoli' size in this image is different from others. However, to respect the inflammatory cells, the size is the same.

2. The correlation between the pulmonary VEGF and alveolar epithelial barrier one side and the role of monocytes on the other side are two possible explanations for the changes of VEGF during VILI. However, the authors work out the differences more detailed in the discussion.

Response:

Thank you for your comments. We are very grateful for your affirmation.

3. In the discussion section (line 378-381) the authors compared the bacterial infection in ventilated patients with their two-hit model of LPS and ventilation. The author should clarify that there is a substantial difference between bacterial infection and LPS treatment in terms of cytokine profiles

Response:

Thank you for your precious comments and advice. We have rewritten the first paragraph (**lines 407-416**) and discussed the substantial difference between bacterial infection and LPS treatment in the second paragraph (**lines 417-428**). In the paragraph, we have discussed the substantial difference of the time-course expression of inflammatory cytokines between bacterial infection and LPS treatment and cited new references (**line 423**).

Reviewer #2: General comments.

Please, clearly decribe how many animals were used in each Group. How many animals entered a Group, how many survived VILI, how many were sacrificed early, etc. Discuss, speculate how this may have impacted on the results? Did animals with an excessive Inflammation die early? Did you check?

Consider a figure, flow chart.

Response:

Thank you for your precious comments and advice. We have made a flow chart in supporting information **S1 Fig**, shown below, to illustrate the numbers of animals used in the different experimental groups and the numbers of animals that died during mechanical ventilation and recovery. For some of the experimental mice died before being sacrificed. The cause of death may have been excessive inflammation and lung injury. This suggests that the concentrations of inflammatory cytokines in the injured lung tissue may have been underestimated. We have stated the situation as a limitation of the study in the discussion section (**lines 541-545**).

S1 Fig. Flow chart depicting animal experiment design.

Please, clearly decribe the ventilation used to induce VILI.

Response:

Thank you for your advice. We have rewritten the section of materials and methods and described the detail of the ventilation set for inducing VILI (**lines 102-111**).

Please, clearly describe the anima handling after the experiment. Did the mice receive pain medication, etc.

Response:

Thank you for your careful review. We have described the detail of the animal handling after the experiment in the section of materials and methods ( **lines 111-115**). The experimental mice did not receive pain medication after the experiment for no tracheostomy creation for intubation and no other wound to be produced by the experimental procedures.

The statistics used may not be appropriate. You used parametric tests despite the low n. The use of non-parametric test would be more cautious. Use non-parametric tests or explain. Did you check for normal distribution in all groups?

Response:

Thank you for your precious comments and advice. We have rechecked the normality of the data by using Shapiro-Wilk test and used parametric or non-parametric tests as appropriate. For comparisons between two groups, data were analyzed by unpaired Mann–Whitney U test. For comparisons between multiple groups, parametric (one-way ANOVA with Bonferroni’s or Dunnett’s post hoc test) or non-parametric test (the Kruskal-Wallis test with Bonferroni correction) was used. Besides, we used Spearmen’s correlation to evaluate the relationship between the IHC intensity of VEGF and the number of Ki67-positive cells in the alveolar epithelium of lung sections (**lines 231-236**). Detailed illustrations of the statistical analysis are provided in the supporting information **S1 Text**.

Avoid nonsensical phrases: The results reveal, etc.

Response:

Thank you for your advice. We have rewritten the phrases (**lines 275 and 379**).

Always state the measurement unit.

Response:

Thank you for your careful review and advice. We have rechecked and made sure that the measurement units have been stated. Besides, for the statements of the IHC staining intensity and mRNA expression, we have presented with relative intensities and fold, respectively.

Line 79

Animals and two-hit model of VILI

The description of the animal model is insufficient.

Clearly describe each hit of the two hit model, e.g.:

The first hit consisted of ……..

The second hit ……..

Then, give a detailed report of the experimental procedure in the correct chronological order.

i. anesthesia

ii. LPS-injection

iii. Intubation

iv. Ventilation

v. Extubation

Specify, the ventilation used to induce VILI besides tidal volume and respiratory rate further: inspiration time, I:E, resulting peak pressure and duration of ventilation!

Whan did you stop ventilation. How and when as a mouse extubated? Did you extubate right from VILI ventilation? What kind of ventilation did you used to wean anesthesia.

Response:

Thank you for your precious comments and advice. We have rewritten and detailed the two-hit model's description in the section of materials and methods (**lines 95-111**).

Line 92:

….water, and the center was maintained in low noises and vibrations.

Sounds awkward. Rephrase

Response:

Thank you for your comment. We have rewritten the paragraph (**lines 89-93**).

Line 112:

The number of animals used per group was as follows: normal control (n = 6), day 0 (n = 6 ), day 1 (n = 6), day 3 (n = 6), day 7 (n = 12, 6 of them for the study of depletion of pulmonary monocytes), and day 14 (n = 12, 6 for the study of depletion of pulmonary monocytes).

This sounds to good. Not one animal died? How many animals did you loose during induction of VILI. How many animals entered each group and how many remained over the following days? What was the selection process to euthanize an animal prematurely?

It needs to be discussed in the discussion section in how far that may have altered the results.

Response:

Thank you for your precious comments and advice. We have detailed in a flow chart, shown in **S1 Fig**, to illustrate the numbers of animals used in the different experimental groups and the numbers of animals that died during mechanical ventilation and recovery. For some of the experimental mice died before being sacrificed. The cause of death may have been excessive inflammation and lung injury. This suggests that the concentrations of inflammatory cytokines in the injured lung tissue may have been underestimated. We have stated the situation as a limitation of the study in the discussion section (**lines** **541-545**).

Line 117:

How were the lungs inflated – manually? With a defined pressure/volume?

Response:

Thank you for your careful review. We have rewritten the section of materials and methods. For histopathology and immunohistochemistry analysis, mouse lungs were manually inflated and fixed by injecting 10% buffered formalin and embedded in paraffin (**line 123**).

Line 215:

How were the mice sedated for this injection. Did you need to ventilate them?

Response:

Thank you for your careful review. We have rewritten and detailed the description of mouse anesthesia in the experiment of “Depletion of pulmonary monocytes” ( **lines 225-226 and 228**). The experimental mice received injection with Clophosome-A in the tail vein after anesthesia with intraperitoneal Zoletil 50. During the experiment, the mice remained with spontaneous breath and did not receive mechanical ventilation.

Line 222:

You used parametric tests despite the small number of animals. Did you check for normal distribution? Non-parametric tests and Spearman correlation may be the more conservative strategy.

Response:

Thank you for your precious comments and advice. We have rechecked the normality of the data and used Spearman correlation to assess association (**line 236**).

Line 245:

C57BL/6 mice received i.v. with 20 ng of LPS and high-stretch mechanical ventilation that produced two-hit VILI.

Sentence does not make sense. Rephrase.

Response:

Thank you for your comment. We have rewritten the sentence (**lines 258-259**).

Line 253:

….; n = 3-5, ….

What does that n mean? It does not match the group size given in the method section?

This refers to all other n = ..... in the figure descriptions.

Response:

Thank you for your careful review. We have correct the typing error in the section of materials and methods ( **line 143**) and removed the wrong description in the section of results (**line 254**). Detailed illustrations of the statistical analysis are provided in the supporting information S1 Text.

Line 262:

Avoid nonsencical phrases like: The results revealed ……

Response:

Thank you for your advice. We have rewritten the sentence (**line 275**).

Line 281:

..SD; n = 4-5,

What is this n?

Response:

Thank you for your careful review. For flow cytometric analysis, the number of animals used per group was as follows: normal control (n = 4), day 0 (n = 4), day 1 (n = 4), day 3 (n = 4), day 7 (n = 5), and day 14 (n = 5), which has been stated in the section of materials and methods. We have correct the descriptions in the legend of Fig 2 (**lines 295 and 299**).

General comment:

What is a background level of something in mice not held under experimental conditions?

If you mean the first measured level before the intervention you may want to use the term baseline. Do you mean the level in the control animals at that specific time point?

Consider using concentration instead of level whenever appropriate.

Response:

Thank you for your precious comments and advice. We have corrected the descriptions to use the term “baseline” instead of “background level” and “concentration” instead of “level” accordingly.

Line 310:

ELISA of inflammatory cytokines and growth factors…..

Specify, lung tissue concentration, plasma concentration?

Response:

Thank you for your careful review. We have detailed the description to clarify that the ELISA samples were obtained from the lung tissue of mice (**line 328**).

Line 323

….from the background level of 252.8 ± 25.9 to 77.3 ± 7.3 on…..

What is the measurement unit? This refers to the entire paragraph!

Response:

Thank you for your careful review and comments. We have correct the measurement unit as the relative staining intensity (**lines 341, 345, 354, and 368**).

Line 328

…. level of 87 ± 5 to 48 ± 5 per 100 alveoli on….

87 what? 87 cells per 100 alveoli?

Response:

Thank you for your careful review. We have correct the description of the unit (**lines 348 and 352**).

Line 354

….VILI (0.19 ± 0.05; p = 0.009, Fig 5) a…

Unit?

Response:

Thank you for your careful review. We have correct the description of the unit (**lines 380, 381, and 383**).

Line 379

….. activated by an underlying bacterial infection.

Rephrase. Bacterial infection is just one of many activators.

Response:

Thank you for your precious comments and advice. We have rewritten the sentence (**line 417**).

Line 391

The expression of Gr1 (Ly6C) on monocytes is closely linked to their divergent roles in inflammatory tissue.

Avoid nonsensical phrases like: linked ….to divergent roles.

Response:

Thank you for your careful review and comments. We have rewritten the sentence (**lines 430-431**).

Line 386

…VILI, contrary to inflammatory cytokines.

That is not contrary. VEGF increases while inflammation fades or vice versa. Rephrase.

Response:

Thank you for your precious comment and advice. We have rewritten the paragraph (**lines 411-412**).

Line 386

VEGF expression in lung tissue is associated with the proliferation of alveolar epithelial cells.

That is an assumption or quote of former works.

First state the main findings. Then, refer to existing knowledge. Then, present a conclusion.

Response:

Thank you for your precious comments and advice. We have rewritten the description that the main finding that the VEGF expression in lung tissue is positively related to the expression of Ki67, a proliferation marker, in alveolar epithelial cells (**lines 412-414**). We have also discussed the finding and possible VEGF roles in post-injury lung referred from other studies (**lines 485-512**). Finally, we conclude that pulmonary VEGF may play a crucial role in post-VILI lung repair (**line 568**).
